# Salvage Reconstruction With Recycled Flap Pedicles in Head‐and‐Neck Surgery: A Report of Two Cases

**DOI:** 10.1002/micr.70141

**Published:** 2025-11-05

**Authors:** Akatsuki Kondo, Hiroki Umezawa, Marie Taga, Rei Ogawa

**Affiliations:** ^1^ Department of Plastic, Reconstructive and Aesthetic Surgery Nippon Medical School Tokyo Japan

**Keywords:** free flap, head and neck reconstruction, pedicle reuse, recipient vessels, salvage surgery, vascularity

## Abstract

Free‐flap reconstruction of head‐and‐neck defects is often complicated by a vessel‐depleted neck after prior surgery or radiotherapy. Conventional alternatives—such as using contralateral vessels, distant recipient vessels, or interpositional vein grafts—are technically demanding and associated with additional risks. We present two salvage reconstructions in which the vascular pedicle of a previously transferred free flap was reused as recipient vessels when standard options were unavailable. A 79‐year‐old man developed exposure of a titanium mandibular plate 6 years after mandibular resection reconstructed with a free anterolateral thigh (ALT) flap. Preoperative ultrasonography and contrast‐enhanced computed tomography confirmed patency of the ALT flap pedicle despite dense fibrosis. After removal of the exposed plate, the pedicle was carefully dissected, and a scapular osteocutaneous flap (9 × 12 cm skin, 2.5 × 11 cm bone) was anastomosed to the lateral circumflex femoral artery and vein of the existing pedicle. Both flaps survived, and postoperative cholecystitis was managed conservatively. In another case, a 63‐year‐old man with a history of reconstruction using a free ALT flap for recurrent temporal meningioma developed another recurrence 2 years later. Imaging confirmed patency of the previous pedicle. During salvage surgery, the pedicle was dissected, and a free rectus‐abdominis flap (9 × 20 cm) was harvested. Arterial anastomosis was performed to the artery of the previous ALT pedicle, and venous drainage was established directly into the internal jugular vein due to insufficient pedicle vein caliber. Intraoperative indocyanine green fluorescence angiography confirmed flap perfusion, and both flaps healed uneventfully. These cases show that reusing the vascular pedicle of a prior free flap may provide a practical salvage option in vessel‐depleted necks. Careful preoperative imaging, intraoperative assessment of flap viability, and meticulous microsurgical technique are essential for success. This approach suggests that pedicle reuse may simplify salvage reconstruction while preserving previously transferred flaps when conventional recipient vessels are unavailable.

## 1 Introduction

Free flap reconstruction in the head and neck critically depends on the availability of suitable recipient vessels. However, prior surgery and radiotherapy can cause vessel depletion due to fibrosis, vessel obliteration, or radiation‐induced vessel wall changes, resulting in a “vessel‐depleted neck.” Common synonyms include “hostile neck” and “frozen neck” (Ad‐El and Sichel 2004; Mulholland et al. 1993; Tan et al. 2014; Abouyared et al. 2019; Kushida‐Contreras et al. 2021).

When recipient vessels are limited, traditional strategies include using contralateral neck vessels, thoracoacromial or internal mammary vessels, or creating arteriovenous loops or vein grafts (Sakurai et al. 2016; Sadove and Kanter 1993; Ribuffo et al. 2004; Xiao et al. 2022).

While these options can be effective, they often require extensive dissection or additional anastomoses, potentially increasing operative time and complications. An alternative strategy is to reuse the vascular pedicle from a previously transferred free flap (Oswald et al. 1988; Pafitanis et al. 2020), though reports of this approach—particularly in head‐and‐neck salvage reconstruction—are rare. We describe two cases where pedicle reuse provided a safe and effective solution when no conventional recipient vessels were available.

## 2 Case Reports

### 2.1 Case 1

**FIGURE 1:**
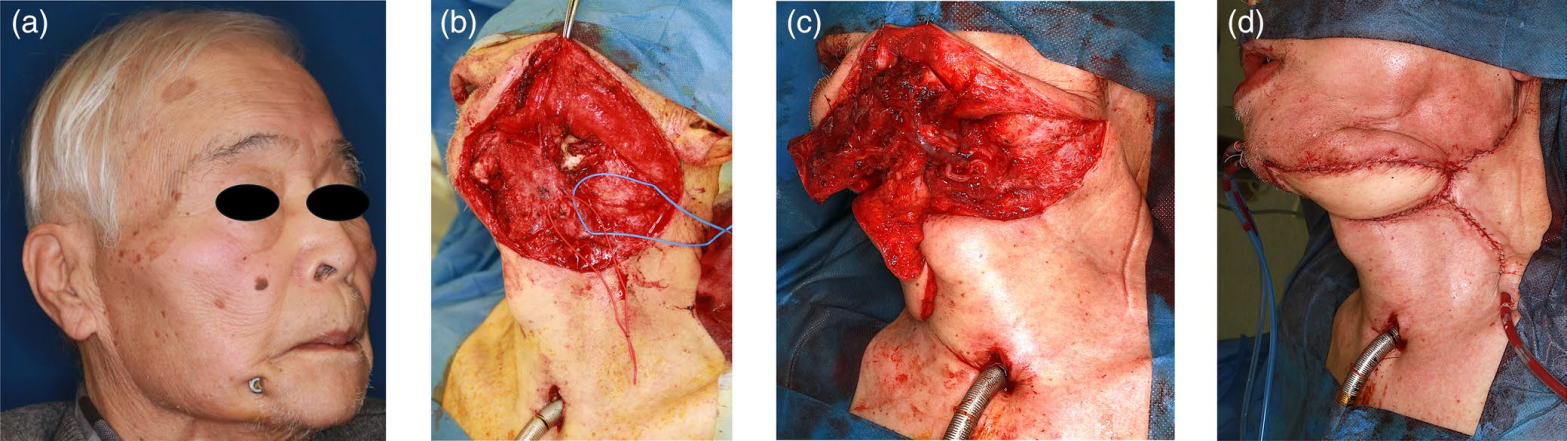
Intraoperative images of Case 1. (a) Preoperative view showing exposure of the mandibular plate on the right side. (b) Dissection of recipient vessels from the previous anterolateral thigh (ALT) flap pedicle following removal of the exposed titanium plate. (c) Microvascular anastomosis of the scapular osteocutaneous flap pedicle to the recipient vessels of the previous ALT flap. (d) Intraoperative view at wound closure.

A 79‐year‐old man had previously undergone segmental mandibular resection and reconstruction for gingival squamous cell carcinoma using a titanium reconstruction plate and a free anterolateral thigh (ALT) flap, anastomosed to the facial artery and vein. Six years later, the plate became exposed (Figure 1a).

Preoperative color Doppler ultrasonography and contrast‐enhanced computed tomography confirmed the patency of the pedicle vessels of the ALT flap, despite extensive fibrosis in the operative field. During salvage surgery, the exposed plate was removed. The pedicle of the ALT flap was carefully dissected through fibrotic tissue to expose the lateral circumflex femoral artery and vein (Figure 1b). To assess the viability of the existing flap, indocyanine green fluorescence angiography was performed after temporary clamping of the pedicle, confirming adequate perfusion of the ALT flap. A scapular osteocutaneous flap with a skin paddle measuring 9 × 12 cm and a bony segment measuring 2.5 × 11 cm was harvested. The flap was inset into the mandibular defect, and microvascular anastomosis was then performed between the scapular flap pedicle and the lateral circumflex femoral artery and vein of the previous ALT pedicle (Figure 1c). Bone fixation was achieved via miniplate fixation and then the wound was closed (Figure 1d). Postoperatively, the patient developed cholecystitis, which was managed with gallbladder drainage and antibiotics. The surgical wounds healed primarily, with no infection or necrosis of either flap. The patient was discharged on postoperative day 34, with restored mandibular contour and oral function.

### 2.2 Case 2

**FIGURE 2:**
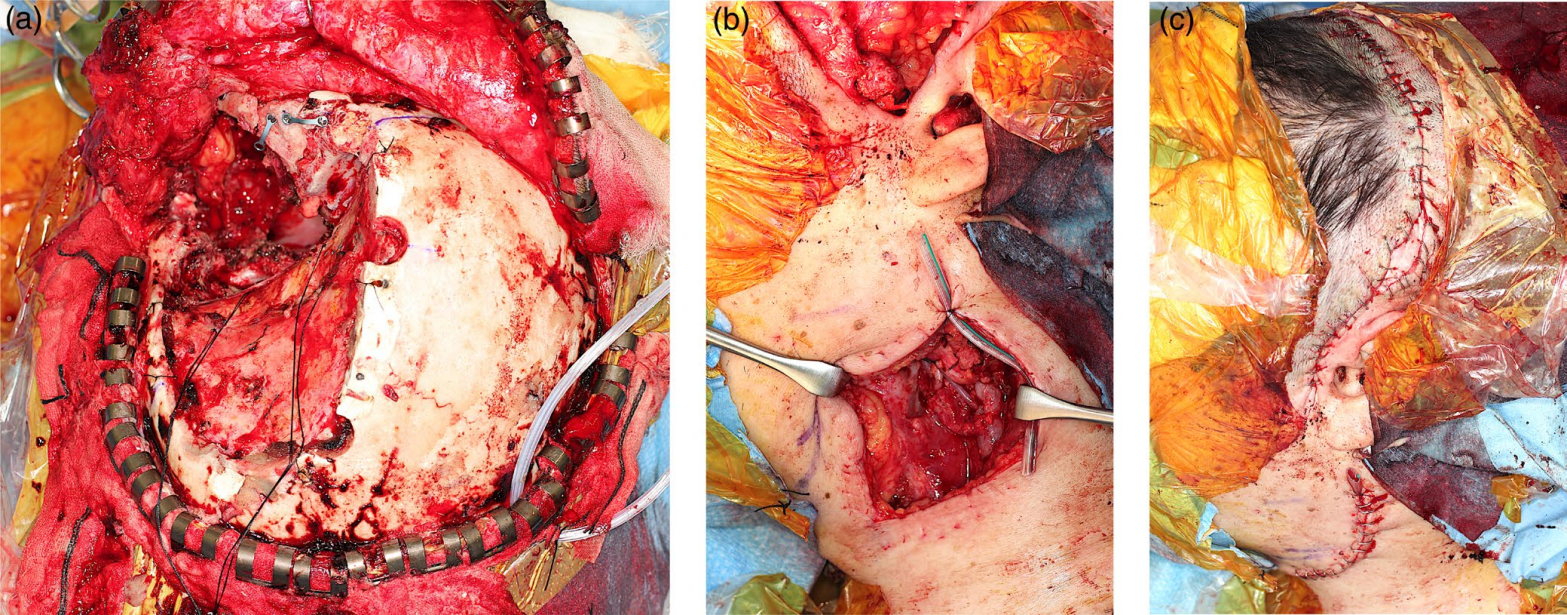
Intraoperative images of Case 2. (a) Defect following tumor resection, with preservation of the previous anterolateral thigh (ALT) flap pedicle. (b) Microvascular anastomosis of the rectus abdominis flap artery to the arterial pedicle of the previous ALT flap. Venous anastomosis was performed to the internal jugular vein. (c) Intraoperative view at wound closure.

A 63‐year‐old man with recurrent meningioma of the left middle cranial fossa underwent tumor resection and reconstruction with a free ALT flap anastomosed to the superior thyroid artery and internal jugular vein. Two years later, the tumor recurred. Preoperative imaging confirmed patency of the previous ALT flap pedicle, while other ipsilateral vessels appeared compromised due to fibrosis. During salvage surgery, the recurrent tumor was removed and the pedicle of the previous ALT flap was carefully dissected (Figure 2a). A 9 × 20 cm free rectus‐abdominis flap was harvested from the left abdomen. The arterial supply of the rectus‐abdominis flap was anastomosed end‐to‐end to the artery of the previous ALT pedicle. As the venous caliber of the pedicle flap was insufficient, venous drainage was established by an end‐to‐side anastomosis to the internal jugular vein (Figure 2b). Flap perfusion was confirmed intraoperatively using indocyanine green fluorescence angiography. Part of the skin flap was externalized, and the wound was closed (Figure 2c). Both flaps survived without congestion or necrosis. The patient was discharged on postoperative day 39 with satisfactory functional and cosmetic outcomes.

## 3 Discussion

These cases show that reusing the pedicle from a previously transferred free flap may be a viable salvage strategy when conventional recipient vessels are unavailable. In vessel‐depleted necks, conventional alternatives can be technically challenging—contralateral neck vessels may be difficult to access, and harvesting distant recipient vessels or creating arteriovenous loops can increase operative time and the risk of thrombosis (Millard 1969; Manrique et al. 2017).

In contrast, pedicle reuse harnesses an already accessible vascular conduit that has proven to be durable. Successful implementation of this approach depends on several considerations. First, preoperative imaging—such as Doppler ultrasonography and contrast‐enhanced computed tomography—should confirm patency of the previous pedicle. Second, intraoperative assessment using indocyanine green fluorescence angiography can verify whether the original flap has developed sufficient neovascularization to survive pedicle ligation. Third, meticulous dissection of fibrotic tissue and precise microsurgical technique are essential to avoid intimal injury; anastomotic sites should be chosen adjacent to, but not directly over, prior anastomoses. Finally, if the venous component of the pedicle is inadequate, an additional venous anastomosis to a nearby recipient vein, such as the internal jugular vein, may be necessary.

Some limitations should be acknowledged. The optimal interval between the initial flap transfer and pedicle reuse remains undefined. In our patients, pedicle reuse was undertaken after 6 years and 2 years, respectively; previous reports suggest neovascularization may develop within weeks, but consistent flap survival is not guaranteed (Fisher and Wood 1984). Moreover, radiation‐induced vessel fragility, dense fibrosis, and patient comorbidities can compromise outcomes (Yoon and Jones 2016; Herle et al. 2015).

Therefore, pedicle reuse may not be applicable in all cases, and careful patient selection is essential. Our experience with two patients suggests that pedicle reuse can reduce operative complexity and preserve the integrity of prior reconstructions, but larger studies are needed to further define its indications and long‐term outcomes.

## Author Contributions

**Akatsuki Kondo:** conceptualization, study design, data collection, surgical procedures, and manuscript drafting. **Hiroki Umezawa:** manuscript review, surgical advice, and critical comments. **Marie Taga:** literature review and minor manuscript editing. **Rei Ogawa:** manuscript review, supervision, and final approval.

## Consent

The patient consented to publishing their case details and photographs with eye masking.

## Conflicts of Interest

The authors declare no conflicts of interest.

## Data Availability

The data that support the findings of this study are available from the corresponding author upon reasonable request.
